# The role of medical regulations and medical regulators in fostering the use of eHealth data for strengthened continuing professional development (CPD): a document analysis with key informants’ interviews

**DOI:** 10.1186/s12909-025-07443-w

**Published:** 2025-07-01

**Authors:** Carol Pizzuti, Cristiana Palmieri, Tim Shaw

**Affiliations:** 1 Department of Medical Education, Melbourne Medical School, The University of Melbourne, Parkville, VIC 3010 Australia; 2 Faculty of Arts and Social Sciences, The University of Sydney, Camperdown, NSW 2050 Australia; 3 School of Medical Sciences, Faculty of Medicine and Health, The University of Sydney, Camperdown, NSW 2050 Australia

**Keywords:** eHealth data analytics, Medical regulations, Continuing Professional Development (CPD) requirements, Medical regulatory bodies, Medical practitioners, Document analysis, Key informants’ interviews

## Abstract

**Background:**

In recent times, medical regulators have been taking measures to strengthen CPD requirements for medical practitioners. In particular, greater emphasis has been placed on CPD activities linked to workplace-based assessment, health outcomes measurement, and quality improvement. These activities require the use of health data, and eHealth data analytics is emerging as a digital solution to simplify tasks and processes. Although there is a growing interest and need for alignment between regulatory policies, impactful CPD activities, and digital health research and innovation, there is little or no research into the role that medical regulations and regulators are playing in fostering the use of eHealth data to strengthen CPD.

**Methods:**

Medical regulations and CPD requirements of 5 selected countries (Australia, Canada, New Zealand, UK, USA) were collected and analysed using the systematic READ approach for qualitative health policy research. Online semi-structured interviews were conducted with 20 key informants from 13 medical bodies to validate findings and gather additional insights. Informants were purposively selected because of their direct involvement in policy development. The interviews were analysed using a hybrid approach of deductive and inductive thematic analysis. The COREQ checklist was used for reporting the findings.

**Results:**

The documents analysed do not mention the use of eHealth data for CPD purposes or refer to it only as a potential data source for CPD completion and compliance. Participants corroborated the document analysis results and provided insights into the following themes: context and rationale of current policy choices and future policy development; roles, responsibilities, and functions of relevant medical bodies in fostering the use of eHealth data for strengthened CPD; barriers, challenges, and enablers for implementation.

**Conclusion:**

Current medical regulations and CPD requirements do not foster the use of eHealth data for CPD purposes. Recommendations for future policy development are reliant on further research on key policy concepts, regulators’ internal organisational factors, and interorganisational collaboration within the CPD ecosystem. The alignment of all relevant CPD stakeholders is required to tackle existing barriers and challenges and promote digital health innovation in the CPD landscape. Medical regulators are called to play a leadership role in this scenario.

**Supplementary Information:**

The online version contains supplementary material available at 10.1186/s12909-025-07443-w.

## Background

In recent times, regulatory medical bodies have been taking measures to strengthen Continuing Professional Development (CPD) requirements for medical practitioners. It has long been recognised that traditional didactic Continuing Medical Education (CME) activities have limited impact on improving clinical practice and patient outcomes [1, 2] and there is increasing consensus on the value of external assessment and feedback in stimulating reflection on practice and encouraging behaviour change [3–6]. Prompted by this, regulators have placed greater emphasis on CPD activities linked to daily medical practice, workplace-based assessment, health outcome measurement, and Quality Improvement (QI) [7–11].

The use of performance feedback and performance data for formative purposes is at the core of workplace-based assessment CPD activities [12]. In particular, several of these activities – such as Audit and Feedback (A&F) interventions, QI projects, and Mortality and Morbidity Meetings (MMM) – require the use of performance data obtained by patient health data analysis [13–15].

While eHealth data is increasingly being used in the delivery of care, large volumes of health data are still contained in paper records [16–18]. In addition, data collection, analysis, and reporting are currently predominantly manual, making the completion of performance work-based assessment CPD activities lengthy and burdensome for medical practitioners [16, 19–21].

In order to address these limitations and to support medical practitioners undertake their CPD, eHealth data usage and big data analytics technologies are emerging as a digital solution to simplify tasks and streamline processes [22–24]. Researchers in both academia and industry are in fact exploring how to make eHealth data more accessible and actionable for health professionals’ reflective practices and CPD obligations [25–29]. Moreover, emerging research is advocating for the integration of information technology systems for learning with clinical information systems to inform health care and to support continuing education and professional development [30].

Although there is a growing interest and need for alignment between regulatory policies, impactful CPD activities, and digital health research and innovation, at present there is no available research on the matter. In particular, there is no clarity on the role of medical regulations and medical regulators in fostering the use of eHealth data for strengthened CPD.

This study aims to address this existing literature gap by investigating the following: (i) if and how eHealth data is referenced/mentioned in current medical regulations and CPD requirements for medical practitioners; (ii) whether eHealth data is mentioned as a data source to complete some of the CPD categories and activities specified in the CPD requirements; (iii) how medical bodies regard the use of eHealth data for strengthened CPD; and (iv) what role these bodies are willing to play with respect to the use of eHealth data analytics technologies for CPD.

## Definition of terms

### Health data and eHealth data

In this study, health data is any personal information concerning the health status, risk, or outcome of an individual and/or pertaining the provision of healthcare interventions and services to the individual [31–34]. eHealth data can be hence considered as health data in digital or electronic format, and therefore includes: Electronic Medical Records (EMRs), Electronic Health Records (EHRs), registries, routinely collected administrative data, claim and billing data, electronic prescriptions, Patient-generated Health Data (PGHD), Patient-Reported Outcome Measures (PROMs), and Patient-Reported Experience Measures (PREMs).

### Regulatory and certifying bodies

Despite the existence of different jurisdictional approaches, the key responsibility of medical regulatory bodies is to protect the public by ensuring that medical practitioners are effectively trained, competent, and fit to practice. In view of this, such authorities are responsible for: (i) establishing a range of measures and systems to assure continued competency among medical practitioners e.g. registration/licensure/certification credentials and processes, (ii) maintaining a public register of practicing medical practitioners, (iii) setting standards of practice for the profession, and (iv) managing complaints and disciplinary actions [35, 36].

Depending on the jurisdiction, some medical regulatory bodies are also responsible for: (1) developing regulatory policies and frameworks for registration renewal, certification renewal/maintenance, or revalidation processes; and (2) setting and enforcing compliance requirements in regard to Continuing Education (CE), CME, and CPD. Among these, CPD compliance requirements are the focus of interest in this study.

Notably, in jurisdictions such as Canada and the USA, some of the abovementioned tasks are carried out by medical certifying bodies. For this reason, these bodies are deemed as relevant for the purposes of this study.

### Continuing professional development (CPD) requirements

Although the literature provides several definitions for Continuing Professional Development [37], in this study CPD refers to those compliance requirements set by the abovementioned regulatory or certifying bodies for registration renewal, certification renewal, revalidation, or maintenance of certification purposes [38].

## Methods

In order to address the research aims of this study, a systematic document analysis of pertinent medical regulations and related CPD requirements for medical practitioners was performed for 5 selected countries. In addition, semi-structured interviews with relevant international key informants were conducted to validate findings and gather additional insights on the matter, as documents may provide a limited description of the context and only a partial account of ongoing debates and potential developments [39].

### Document analysis

Document analysis is a qualitative research method, and it can be defined as a “systematic procedure for reviewing or evaluating documents – both printed and electronic” [40] (p. 27). Its results are often triangulated with other sources of data, such as interviews and academic literature, to contextualise findings, seek corroboration, and gather additional insight [40, 41].

The document analysis illustrated in this study was undertaken using the 4-step systematic method for qualitative health policy research named the READ approach: (1) Ready your materials; (2) Extract data; (3) Analyse data; and (4) Distil your findings [42].

In a preliminary phase, Australia, Canada, New Zealand, the UK, and the USA were purposively selected as countries of interest because of their historical relations, ongoing collaboration, and mutual influences in terms of health policy development and implementation.

Subsequently, (i) relevant bodies for each country were selected based on their role and responsibilities in setting CPD requirements, and (ii) related medical regulations and CPD requirements were identified and stored in NVivo (Version 20.6.1, from QSR) for data extraction and analysis (step 1). The documents deemed pertinent are all in the public domain, and they are freely available on the internet as downloadable files or webpages (Table 1).

**Table 1:** List of relevant bodies and corresponding documents for analysis

Country	Body	Document	Type	Link
Australia	MBA	Registration	Webpage	https://www.medicalboard.gov.au/Registration.aspx
		Registration standard: Continuing professional development	PDF	https://www.ahpra.gov.au/documents/default.aspx?record=WD21%2F31046%26dbid=AP%26chksum=TqPI98CYQYllvPkGwiAz%2Fw%3D%3D
Canada	CFPC	Mainpro + Manual	PDF	https://www.cfpc.ca/en/Resources/Continuing-Professional-Development/Mainpro-Manual
	Royal College	The Maintenance of Certification Program	Webpage	https://www.royalcollege.ca/en/cpd/moc-program
		The MOC framework	Webpage	https://www.royalcollege.ca/en/cpd/moc-program/moc-framework
	CMQ	Règlement sur la formation continue obligatoire des médecins	PDF	https://www.legisquebec.gouv.qc.ca/fr/pdf/rc/M-9,%20R.%2022.1.pdf?t=1669607018480
		Les obligations des médecins en matière de formation continue	PDF	http://www.cmq.org/publications-pdf/p-1-2018-11-29-fr-obligations-medecins-formation-continue.pdf
New Zealand	MCNZ	Recertification and professional development	Webpage	https://www.mcnz.org.nz/registration/maintain-or-renew-registration/recertification-and-professional-development/
		Policy on recertification programmes for doctors – August 2021	PDF	https://www.mcnz.org.nz/assets/Policies/dc6f03cda4/Policy-on-recertification.pdf
		Recertification requirements for vocationally-registered doctors in New Zealand – November 2019	PDF	https://www.mcnz.org.nz/assets/Publications/Booklets/f7d4bc7fff/Strengthened-recertification-requirements-for-vocationally-registered-doctors-November-2019.pdf
UK	GMC	Guidance for doctors’: requirements for revalidation	Webpage	https://www.gmc-uk.org/registration-and-licensing/managing-your-registration/revalidation/guidance-for-doctors-requirements-for-revalidation
		Guidance on supporting information for revalidation guide	Webpage	https://www.gmc-uk.org/registration-and-licensing/managing-your-registration/revalidation/guidance-on-supporting-information-for-revalidation
		Continuing professional Development. Guidance for all doctors	PDF	https://www.gmc-uk.org/-/media/documents/cpd-guidance-for-all-doctors-0316_pdf-56438625.pdf
USA	ABMS	Board Certification Standards	Webpage	https://www.abms.org/board-certification/board-certification-standards/
		Board Certification Requirements	Webpage	https://www.abms.org/board-certification/board-certification-requirements/#cc
		Standards for Continuing Certification	PDF	https://www.abms.org/wp-content/uploads/2021/11/ABMS-Standards-for-Continuing-Certification-20211029.pdf

Descriptive information was then extracted and analysed using basic qualitative content analysis [43] (step 2 and 3). Data was extracted on the following matters: (1) medical regulatory systems; (2) CPD requirements and their effective date; (3) CPD categories, as set by the relevant medical body and specified in the corresponding CPD requirements; (4) CPD categories and activities that need the use of health data to be completed; (5) mention of the use of eHealth data for the completion of the categories and/or activities in point 4.

Finally, findings were distilled into a tabular form and also into a descriptive format (step 4).

### Key informants’ interviews

Semi-structured interviews with key informants were conducted in order to validate the findings of the documentary analysis and collect additional information on policy development and implementation.

As a first step, an environmental scan was conducted to identify potential participating medical bodies. The definitions of regulatory and certifying body provided above were used as a discriminant for determining whether the body was suitable for the purposes of this study. Subsequently, executives, managers, and administrators directly involved in the development of medical regulations and the establishment of CPD requirements were contacted using general administration or individual email addresses depending on the information publicly available on the internet. Of the 27 regulatory and certifying bodies contacted, 13 agreed to participate (Table 2). In total, 37 individual email addresses and 28 generic email addresses were used to reach potential research participants, resulting in interviews with 20 key informants from the 13 participating bodies (Table 2).

**Table 2:** List of the research participating bodies and number of research participants per country

Country	Body	Type of Body	Number of Participants
Australia	Australian Health Practitioner Regulation Agency (Ahpra)	Regulatory Body	5
	Medical Board of Australia (MBA)	Regulatory Body	
Canada	Federation of Medical Regulatory Authorities of Canada (FMRAC)	Regulatory Authority	5
	College of Physicians and Surgeons of Manitoba (CPSM)	Regulatory Authority	
	The Royal College of Physicians and Surgeons of Canada (Royal College)	Certifying College	
	The College of Family Physicians of Canada (CFPC)	Certifying College	
	College du medicine du Quebec (CMQ)	Licensing and Certifying College	
New Zealand	Medical Council of New Zealand (MCNZ)	Regulatory Body	2
UK	General Medical Council (GMC)	Regulatory Body	2
USA	American Board of Medical Specialties (ABMS)	Certifying Board	6
	American Board of Surgery (ABS)	Certifying Board	
	American Board of Ophthalmology (ABO)	Certifying Board	
	Accreditation Council for Continuing Medical Education (ACCME)	Non-profit Regulatory Corporation	

To note, some bodies had multiple key informants involved in developing medical regulations and CPD requirements. As a result, more than one key informant was interviewed from certain regulatory and certifying bodies, provided they made themselves available for interview.

Interviews were conducted following a semi-structured outline approved by the University of Sydney Human Research Ethics Committee (Protocol Number 2020/722). The interview questions focused on the following matters: (i) national regulatory landscape; (ii) content of medical regulatory documents and CPD requirements; (iii) role of the body in regulating CPD; (iv) health and eHealth data analytics and CPD requirements; (v) role of the body in fostering the use of eHealth data analytics for CPD purposes.

All interviews were conducted on Zoom; audio-recordings were transcribed verbatim using DeScript. After being assessed for accuracy, interview transcripts were stored in NVivo and analysed using a hybrid approach of deductive and inductive thematic analysis [44, 45].

At first, broad a *priori* codes were established by CPi, drawing from the study aims and the semi-structured interview outline. As the analysis progressed, additional *a posteriori* codes were developed from the interview data. To ensure alignment and reduce bias in the a posteriori coding, three transcripts were independently coded by CPi and CPa. The coding was then compared to establish intercoder agreement and reliability.

CPi subsequently completed the analysis of the remaining transcripts, with iterative coding conducted until data saturation was reached.

Once the analysis was finished, CPi and CPa reviewed, combined, and finalized all the codes through discussion and reflected on the interpretation of the results. There were no disagreements regarding the final coding or interpretation.

The Consolidated Criteria for Reporting Qualitative Research (COREQ) checklist [46] was used to ensure accurate and complete reporting of the interview findings.

## Results

### Document analysis results

Given the variance of regulations, stakeholders, and terminology across the jurisdictions under investigation, document analysis results are presented alongside with an overview of the medical regulatory system and processes for each country to assure clarity.

A detailed account of data extraction results is provided in Table 3.

**Table 3:** Document analysis: data extraction results

Country	AU	CAN	CAN	CAN	NZ	UK	USA
**Type of Body**	Medical Regulatory Body	Certifying College	Certifying College	Licensing and Certifying College	Medical Regulatory Body	Medical Regulatory Body	Medical Regulatory Body
**Name of Body**	Medical Board of Australia (MBA)/ Australian Health Practitioner Regulation Agency (Ahpra)	The College of Family Physicians of Canada (CFPC)	The Royal College of Physicians and Surgeons of Canada (Royal College)	Collège des médecins du Québec (CMQ)	Medical Council of New Zealand (MCNZ)	General Medical Council (GMC)	American Board of Medical Specialties (ABMS)
**Medical regulatory systems**	Registration Standards	Maintenance of Certification	Maintenance of Certification	Maintenance of Certification	Recertification Programme	Revalidation	Continuing Certification
**CPD requirements**	CPD Registration Standards	Mainpro+^®^ (Maintenance of Proficiency) Program	Maintenance of Certification (MOC) Program	Formation continue obligatoire des Médecins	CPD Requirements	CPD Requirements	Standards for Continuing Certification
**Effective**	From 1 January 2023	From 27 June 2016	From January 2024	From 1 Jan 2019	From 1 July 2022	From June 2012	From 1 Jan 2024
**CPD categories**	3 Categories: 1. Educational activities 2. Reviewing performance activities 3. Measuring Outcomes activities .	3 Categories: 1. Group learning 2. Self-learning 3. Assessment activities Within those categories, activities can be either certified or non-certified.	3 Sections: 1. Group learning 2. Individual learning 3.Feedback and improvement	3 Activities: 1. Activités de développement professionnel (DP) reconnues 2. Activités d’évaluation de l’exercice (EE) reconnues 3. Activités de formation continue non reconnues mais admissibles	3 categories: 1. Reviewing and reflecting on practice 2. Measuring and improving outcomes 3. Educational activities (continuing medical education – CME)	No prescription of a specific number of CPD credits or hours.	3 elements: 1. Professional Standing and Conduct 2. Lifelong Learning 3. Improving Health and Health Care
**Health data use and CPD categories**	Not Applicable, as the MBA CPD Registration Standards do not provide descriptions/definitions for CPD Categories.	Category 2: Self-assessment Category 3: Assessment	Section 1: Group learning Section 3: Feedback and improvement	Activité 2: Évaluation de l’exercice	Category 2. Measuring and improving outcomes	Not Applicable, as the GMC does not set CPD categories.	Element 3: Improving Health and Health Care
**Health data use and CPD activities**	Not Applicable, as the MBA CPD Registration Standards do not provide a list of activities for each Category.	‘Linking Learning to Practice” activity *(Category 2*) Practice audits Quality assurance programs Provincial practice review and enhancement programs *(Category 3*)	Morbidity and mortality rounds (*Section 1*) Annual performance reviews Chart audits Reviews of clinical data Quality Improvement (QI) (*Section 3*)	Activités d’évaluation de l’acte médical	“Quality improvement process that includes review (internal or external) of a doctor’s everyday work and resultant patient/health outcomes.”^1^ (p. 6)	Audits Quality improvement activity *(Revalidation requirement)*	Quality improvement activities
**Mention of eHealth data**	eHealth data not mentioned	eHealth data not mentioned	eHealth data not mentioned	Electronic Medical Records (EMRs) as a potential data source	eHealth data not mentioned	eHealth data not mentioned	eHealth data not mentioned

^1^ Medical Council of New Zealand (MCNZ). Recertification requirements for vocationally-registered doctors in New Zealand. November 2019. Available at: https://www.mcnz.org.nz/assets/Publications/Booklets/f7d4bc7fff/Strengthened-recertification-requirements-for-vocationally-registered-doctors-November-2019.pdf. Accessed December 6, 2022

### Australia

In Australia, the Australian Health Practitioner Regulation Agency (Ahpra) regulates registered health practitioners, and it works in collaboration with 15 National Boards to set the standards that all health practitioners must meet to register and maintain their registration to practice medicine [47]. In particular, the Medical Board of Australia (MBA) sets professional regulation and registration standards for medical practitioners in Australia [48], in accordance with the Health Practitioner Regulation National Law [49]. Except for registered students and non-practising registrants, MBA mandatory registration standards apply to all applicants for registration and registration renewal [50]. Among other standards, the MBA sets the CPD Registration Standards for all Australian medical practitioners.

As per Table 3, the current MBA CPD Registration Standards do not mention the use of eHealth data for CPD purposes.

Available supporting documentation indicates though that: (i) both health and eHealth data are at the basis of “reviewing performance” and “measuring outcomes” activities [51] ( p. 9); (ii) “improved access to data will help improve safety and quality by giving doctors the tools to reflect on and review their practice routinely and measure outcomes” [52] (p.3); and that the MBA will “urge governments and other holders of large data (such as Medicare) to make data accessible to individual registered medical practitioners to support performance review and outcome measurement” [52] (p. 7).

### Canada

In Canada, provincial and territorial Medical Regulatory Authorities (MRA) are responsible for registering and licensing all medical practitioners who practice within their jurisdiction. These authorities, some of which are also known as “licensing colleges”, are members of and report to the Federation of Medical Regulatory Authorities of Canada (FMRAC) [53].

Canadian medical practitioners must meet the registration requirements set by the relevant MRA to achieve full licensure and must obtain a certification by examination before applying for medical registration [54].

In this regulatory landscape, CPD is an essential component of license renewal, maintenance of certification, admission and renewal of college Fellowship, and attainment and maintenance of college post-nominal designations.

MRA-approved CPD programs are offered by the Canadian certifying colleges i.e., The College of Family Physicians of Canada (CFPC), The Royal College of Physicians and Surgeons of Canada (Royal College), and the Collège des médecins du Québec (CMQ). Every certifying college sets different CPD requirements for their fellows, offers a different CPD program, and establishes different CPD categories. Apart from these differences, what is of interest for the purposes of this study is that health data is one of the potential data sources necessary to complete the activities in the “assessment” category in all CPD programs. However, eHealth data is not explicitly mentioned in the programs, or it is referred to as a potential data source only. For comprehensiveness, it is necessary to report that the CFPC developed an initiative that refers to health data and eHealth data usage for CPD purposes.

In 2017, in fact, the CFPC launched the Practice Improvement Initiative (Pii) to promote the use of “quality improvement, practice-level data, and research to improve everyday practice” [55] in family medicine. The Pii is categorized as an “advocacy and innovation” initiative and states that practices and primary care teams are called to “promote Mainpro+^®^ credits [CPD credits] for activities related to QI and the use of practice-level data” [55].

### New Zealand

Under the Health Practitioners Competence Assurance Act 2003 (HPCAA) [56], New Zealander medical practitioners must be registered with the Medical Council of New Zealand (MCNZ) and hold a current practicing certificate to practice medicine. To maintain their right to registration and demonstrate competence to practice, they must also meet the MCNZ recertification program requirements [57] – which include participating in Continuing Professional Development [58].

Current MCNZ CPD requirements are specified in the policy document titled “Recertification requirements for vocationally-registered doctors” [59]. In terms of health data usage, the policy states that “patient/health outcomes” are necessary to complete activities that fall in the “Measuring and improving outcomes” CPD category. However, there is no mention of eHealth data usage for such purpose.

### UK

In the UK, medical practitioners need to be registered with and licensed by the General Medical Council (GMC) in order to practise medicine [60]. Starting from 2012, all medical practitioners who hold registration with a licence to practise in the UK must comply to revalidation requirements [61].

Revalidation is a mandatory 5-year process that licensed practitioners must undertake to demonstrate that they meet the principles and values set out in the “Good medical practice” guidance [62], and it is defined by the GMC as “an evaluation of [medical practitioners’] fitness to practise” [7] (p. 3). Among other obligations, revalidation includes also annual CPD requirements.

Regarding the use of health data, tools like audits and QI initiatives are repeatedly mentioned in the GMC CPD guidance [63]. Also, the revalidation process itself includes the requirement to participate in at least one documented QI activity in every cycle [7]. Although these tools and activities require the analysis of patient-related data, there is no mention of eHealth data usage in the GMC policy documents.

### USA

In the USA, medical practitioners receive a licence to practice medicine in a particular state after completing medical school and passing a licensing exam. After residency, practitioners may choose to become board certified. Board certification validates practitioners’ competency standards and serves as a reliable indicator of the practitioner’s commitment to life-long learning, ongoing practice improvement, and excellence in patient care. Once certified, practitioners must undertake continuing certification programs offered by relevant medical certifying boards in order to retain their certification [64].

The American Board of Medical Specialties (ABMS) is responsible for setting professional standards for both initial and continuing certification in partnership with its 24 Member Boards, whereas CPD frameworks and programs are to be set and offered by the Member Boards depending on medical specialty.

Bearing in mind the complexity of the CPD landscape in the USA, what is of interest for this study is that the QI activities mentioned in the element “Improving Health and Health care” of the ABMS Standards for Continuing Certification [10] require the use of health data to be completed. However, eHealth data is not explicitly mentioned in the Standards.

## Key informants’ interviews results

The key informants confirmed and corroborated the findings discussed in the “Document Analysis Results” section with regard to: (1) regulatory landscape, systems, and processes; (2) CPD requirements, and (3) CPD categories and activities, and health/eHealth data usage for category and activity.

In addition to providing validation of results, informant interviews offered nuanced insights that extended beyond the scope of the document analysis itself. They in fact served a role in elucidating the present policy rationale, forecasting potential future policy trajectories, and unveiling both current and prospective intentions regarding policy implementation, decision-making processes, and the overarching regulatory landscape within the realm of eHealth data analytics usage for CPD purposes.

Specifically, the analysis of the interviews led to the identification of the 3 main themes described below. Full and additional quotes are provided in Additional File 1.

Considering the nature of the interview analysis’ results, traditional data triangulation and comparison posed significant challenges and has not been undertaken in this study.

### Theme 1: use of eHealth data for strengthened CPD

*Valuable but complex.* Several participants shared their opinion on the use of eHealth data for CPD compliance, describing it as a valuable opportunity with great potential for practice improvement and continuous learning and development: “*I think there’s huge potential. […] we’d like to see a world where practitioners are supported to continue to improve and to learn and develop and grow*,* and data is such a fantastic way for that to happen.*” (P15, Australia).

However, many participants also commented on the complexity of such endeavour, pointing out how practices, processes, and procedures of performance assessment and measuring health outcomes are still in their infancy, and mentioning the existence of several barriers and challenges for the implementation of eHealth data analytics for CPD purposes.

*Barriers.* All participants stated that the biggest barrier to using eHealth data for CPD purposes is related to data availability and accessibility: “…*we actually understood that*,* that it might be difficult [for medical practitioners] accessing data for some of the things that they need to do*,* so it’s not*,* we know it’s not at the moment readily available. We know this is a work in progress*” (P13, Australia).

Lack of mature data systems and/or data system standardization, poor interoperability between existing data systems, and great variance in terms of data accessibility depending on jurisdiction, location, settings, and specialty were reported as the main issues to address for successful implementation.

In addition, participants’ concerns related to eHealth data usage for CPD purposes included – but were not limited to – ongoing controversies around data privacy, data sharing, and data relevance, and current limitations in terms of data quality, data integrity, and meaningful data reporting.

*Challenges.* All participants pointed out that current systemic issues around stakeholders’ priorities, strategic planning, and coordination of roles and responsibilities represent the main challenge for the implementation of eHealth data analytics for CPD compliance. Such position could be summarised as follows: “*…there are no plans. There’s not even a group that’s looking at it. But there’s been a lot of talk and recommendations*,* but no one’s actually rolling up their sleeves to move this along. […] There’s no national coordination of this*,* right? It’s left to like*,* I think the hospitals… […] I think if we were to get together and say*,* ‘Here’s the greater good*,* and here’s what it looks like.’ And we paint the picture of what we really want to enable*,* then we could divide off the roles. […] we would do it in a coordinated way that may actually contribute to a cohesive system. But that’s what’s missing.*” (P06, Canada).

In addition to this, a number of additional challenges were mentioned by the informants: (i) medical practitioners’ general attitudes around professional self-regulation and CPD value and effectiveness, (ii) medical practitioners’ concerns around confidentiality, privacy, ownership, and potential punitive use of individual performance data, and (iii) lack of workforce capabilities in engaging in QI activities and in using eHealth data analysis for such purposes.

*Enablers.* Although not prompted, participants suggested enablers and facilitators to foster the use of eHealth data for CPD purposes: (i) making use of champions in healthcare service organisations in order to build trust among medical practitioners and promote the adoption of eHealth analytics practices at organisational, team, and individual level; (ii) building a “support system” embedded in the workplace through the employment of advisory groups, mentors, and facilitators to assist teams and individuals in interpreting their data and making it actionable; (iii) making more extensive use of Artificial Intelligence (AI) and machine learning at healthcare service level to avoid manual analysis and reporting, additional work for medical practitioners, and, consequently, potential workforce burnout.

### Theme 2: eHealth data and policy

When discussing the results of the document analysis, several participants provided rationale for current policy choices and future policy development, and shared insights on policy development processes and policy terminology.

*Current policy choices and future policy direction*. Key informants clarified the motives for the lack of mention of eHealth data in current policy documentation. Their reasons varied from emphasising that regulators mostly provide high-level guidance with regard to CPD requirements and that the use of eHealth data for CPD is at the discretion of the individual medical practitioner, to commenting that prescribing the use of eHealth data could be a limitation rather than an opportunity. Some representative quotes include the following:

“*It’s actually not for us to say you have to use this set of data.*” (P13, Australia).

*“…we’d say what data would you find useful and how does that shine a light on your practice*,* and how does that help you reflect on what you’re doing*,* and you choose the data source that is most appropriate for that activity.*” (P19, UK).

“*I would find it unlikely that we would say it must be this way with the electronic health record or with anything because that’s sort of […] very limiting for the diplomates who were engaged in a lot of different efforts to improve care.*” (P05, USA).

Notably, some participants also shared their insights on future policy directions, clearly stating whether eHealth data usage will be made prescriptive or not:

“*We won’t prescribe it.”* (P19, UK).

*“We’re changing our regulation of medical records outside of hospital*,* and it’s submitted to the [governmental organisation] because we want the regulation to clearly stipulate that every physician in Canada will have to use an EMR. No more of those chart handling*,* and paper*,* and everything*,* and the loss*,* and the whatever*,* finished. […] Everybody will have to use an EMR to evaluate their practice.”* (P08, Canada).

*Current policy intent.* A few research participants shed light on policy development processes, declaring that any mention of eHealth data and eHealth data analysis in policy documents does not imply the use of digital technologies nor big data analytics. Rather, it is an acknowledgment of eHealth data as a potential data source and of eHealth data analytics as a potential opportunity. As some participants stated: “…*in the discussions that we had at the CPD expert group that developed the* [CPD framework], *people weren’t attuned to the idea that there would be this sophisticated analytics. […] That there was recognition that there were these data collected but not a clear sense of how that might be used other than with those certain manual extraction type of way. […] It wasn’t in the clear intentions*,* but the potential was by no means excluded.*” (P03, Australia).

*Meaning of term “data”.* Some key informants argued that the term “data” in policy documents and CPD requirements does not necessarily refer to patient health data nor to eHealth data. Rather, “data” indicates any piece of information pertaining to medical practitioners’ practice and/or performance: “*We think much more broadly around this whole reflecting on outcomes*,* um*,* because we are using data in a much broader sense. We’re talking about data from multi-source feedback*,* outcomes data from visits to your practice and*,* and different things outside the normal thought pattern around data*.” (P01, Canada).

### Theme 3: Roles and responsibilities of medical bodies

All key informants expressed agreement on the potential advocacy and lobbying function of medical regulatory and certifying bodies in fostering the use of eHealth data for CPD compliance. Governments, the private healthcare sector, and/or any agency or organisation that regulates eHealth data management and governance at any level (national, provincial, state, territorial, organisational) were mentioned as relevant stakeholders to liaise with. As some participants remarked: “*It’s our job to form those alliances*,* form those partnerships with a whole range of different stakeholders*,* and all be pulling in the one direction to help practitioners to continuously improve.*” (P4, Australia).

Also, key informants suggested that relevant medical bodies should take the following responsibilities to address current implementation barriers and challenges: (i) engaging in partnerships with external data experts and research groups to link eHealth data analytics to medical regulatory processes and/or to receive guidance and recommendations on how to proceed in that regard; (ii) for those bodies that also offer CPD resources, providing information, education, and support to medical practitioners with regards to eHealth data access and actionability; (iii) for those bodies that also accredit CPD providers, rewarding the organisations that emphasise the usage of eHealth data in their programs and activities.

## Discussion

The purpose of this study was to analyse current medical regulations and related CPD requirements for medical practitioners in 5 selected countries in order to understand whether relevant policy documents encourage the use of eHealth data for CPD completion and compliance. Also, the study aimed to explore the standpoint of medical regulatory and certifying bodies on the matter, and to clarify what roles these bodies are willing to play to foster the use of eHealth data analytics for strengthened CPD.

The academic field of health professionals’ regulation is relatively underdeveloped [65] and, in particular, systematic evidence on the development of CPD requirements based on patient health outcomes is lacking [66]. Considering this, the discussion provides a summary of the results, attempts to provide a commentary in the context of relevant scholarly research, and proposes some recommendations for further research and future policy development and implementation.

### Current policy choices: considerations on policy rationale and recommendations for further research

The overall results of the study show that medical regulations and CPD requirements do not currently foster the use of eHealth data for CPD purposes.

From the documents analysed, health data is generally considered necessary: (i) for the completion of those CPD categories that focus on performance assessment and/or review and/or aim at measuring and improving health outcomes; and, in particular, (ii) for the completion of CPD activities undertaken at the workplace and strongly linked to daily medical practice, such as audits and QI initiatives. However, the use of eHealth data for the same purposes is not explicitly mentioned in current CPD requirements for medical practitioners. The only exception is the CMQ “Formation continue obligatoire des Médecins”, which refers to Electronic Medical Records (EMRs) as potential data sources for CPD completion (Table 3).

It is important to note that Australian supporting documents and the CFPC Pii do allude to the employment of eHealth data analysis for performance assessment and QI CPD activities [8, 55]. However, the content and the intention of the MBA supporting documents is not reflected in its corresponding CPD Registration Standards, and the principles of the CFPC Pii are not yet fully integrated in the CFPC requirements around CPD compliance.

Considering the interview results, it appears that that three main determinants influence current policy choices: (1) contextual barriers and challenges, (2) the high-level regulatory function of medical regulatory bodies, and (3) the comprehensiveness of the term “data”.

With regard to the first determinant, informants’ observations are supported by other research. Participants’ concerns around major data barriers are in fact in line with the existing literature on clinical performance and health outcome measurement issues [67–69], challenges with post-implementation experiences of large-scale EHRs [70], and clinical data quality and accuracy [71, 72]. Also, participants’ comments on the presence of systemic, organizational, and individual challenges in the CPD landscape appear to reflect the findings on ongoing research on the associations between cultural and systemic factors and the use of clinical data for life-long learning (LLL) and CPD [73, 74], biomedical and health informatics (BMHI) education for health professionals [75, 76], and health practitioners’ attitudes towards and engagement with the use eHealth data for CPD purposes [77, 78].

In light of this, participants’ standpoint could be considered as well-informed and regulators’ policy choices regarded as reasonable. However, it appears that regulators are currently overlooking ongoing industry and scholarly research in the fields of learning and practice analytics applied to performance assessment practices across the health professions education continuum [25, 30, 79–87]. Both fields are providing evidence on how performance data obtained by eHealth data analysis can be used to prompt self-reflection, identify knowledge and skill gaps, and better educational curricula and programs with the ultimate aim of improving clinical practice and patient care.

Taking into account these latest research developments, document analysis results would suggest that most regulators are yet to reflect digital health innovation research and trends in their regulatory policies and CPD requirements.

It is important to note, though, that some medical bodies are showing a different approach in this regard, with some participants expressing the intention to include eHealth data in future policies or even mandate its use for CPD compliance purposes. For this reason, it is necessary to conduct more research on organizational culture, vision, and priorities, legislative environment, and other potential jurisdiction-specific factors to understand whether these elements are having an impact on regulators’ decision and policy making processes in regard to the use of eHealth data for CPD.

In relation to the second determinant, the majority of the informants stated that medical regulatory bodies have the institutional responsibility to set CPD requirements but not the authority to establish detailed regulations in this regard nor the onus to indicate or prescribe what type of data sources should be employed for CPD completion. However, considering that the interviews show contradicting results on the matter and that there is little research examining the effectiveness of current regulatory practices [88], it would be beneficial to investigate the subject further. In particular, there would be value in exploring the point of view of other stakeholders (e.g., CPD providers, professional associations, healthcare service organisations, governmental agencies) in order to clarify expectations around the CPD regulatory functions of the regulators.

Finally, the third policy determinant is related to the conceptualization of the term “data” in policy documents and CPD requirements. Interview results show that medical regulators and certifying bodies give a broad meaning to the concept of “data” for performance assessment and QI purposes, regarding it as any piece of “information, especially facts and numbers, collected to be examined and considered and used to help decision-making” [89] rather than “information in an electronic form that can be stored and processed by a computer” [89].

Using the first definition as a reference, performance assessment “data” might thus include colleagues and patients’ questionnaire results from Multi-Source Feedback (MSF) tools [90, 91] or reviews by peers and/or non-physicians observers from practice visits [92, 93].

It is crucial to clarify though that the assessment outcomes of tools such as MSF and practice visits are based on the *feedback* of colleagues, patients, and/or external observers whereas the assessment outcomes of tools such as MMM, clinical audits, and QI projects are largely based on the retrospective and/or prospective analysis of *patient health data* (in paper and/or electronic format) [15, 94, 95]. Despite this core difference, at present scholarly literature often presents the terms “data” and “feedback” as interchangeable [96] and does not provide an explicit and shared definition of “individual performance assessment data”.

Considering the lack of clarity and consensus on these terms, it would be advisable for medical bodies and academia to collaborate in the formulation of key concepts for both future policy development and research advancement.

### Future policy prospects: regulators’ intentions, missed opportunities, and considerations on policy development and implementation

The overall results of the interviews provide discrepant evidence in terms of future policy development and implementation to foster the use of eHealth data for strengthened CPD.

Key informants had different opinions as to how to proceed in this regard, and their positions around the use of eHealth data for CPD compliance sat on a continuum that ranges from discretionary to mandatory – with the majority of participants agreeing that the regulatory and certifying bodies will not include nor prescribe eHealth data usage in policy documents and CPD requirements in the foreseeable future. Furthermore, considering that none of the participants explicitly mentioned policy development and implementation as one of the current or potential responsibilities of medical regulators, policy development prospects appear uncertain.

Nevertheless, the development of clear regulatory policies around the use of eHealth data for strengthened CPD could lead to a number of opportunities for the regulators, including: (1) contributing to the ongoing conversation on performance-based assessment strategies and data-driven CPD [97]; (2) taking an active role in encouraging digital health innovation in CPD practices; (3) clarifying the formative use of eHealth data and the educational value of its analysis so to address medical practitioners’ concerns around potential summative actions in case of poor performance outcomes [73, 77]; (4) prompting other key stakeholders in the CPD ecosystem to engage with the current debate, consider their own role in fostering eHealth data for CPD purposes, and assume responsibilities to support implementation.

Given this, a call for medical regulators to develop policies and implementation strategies to foster the use of eHealth data for CPD may seem obvious. The jurisdictional variance in terms of regulatory systems and relevant stakeholders and the need for further research on key concepts, internal organisational factors, and interorganisational dynamics do not support the development of generalisable yet specific recommendations at this point in time. However, the following considerations could inform policy decision-making for all relevant medical bodies: (1) the importance of making policy intentions clear with regard to the regulators’ expectations of eHealth data usage and analysis, and (2) the necessity of including a precise definition for the term “data” and other key concepts in regulatory policies and relative supporting documents.

This is particularly relevant especially because multiple stakeholders with different expertise, interests, and objectives will be eventually involved in policy implementation. As an example, stakeholders in the digital health research space focus on the use of patient eHealth data and of digital technologies to improve clinical practice and healthcare [98, 99]. The current ambiguity of what constitutes “data” in policy documentation might lead to miscommunication and misunderstandings with digital health experts, and therefore to misalignment in terms of research aims and outcomes, and expectations around deliverables and recommendations for future implementation.

### Medical regulators and the fostering of eHealth data strengthened CPD: unfulfilled functions, areas of influence, and suggested role

In terms of the role of medical bodies in fostering the use of eHealth data for strengthened CPD, study findings reveal a current paradoxical situation. Even though key informants agreed that the main function of medical regulators is to address data accessibility and availability barriers through advocacy and lobbying, overall study results indicate that this remains largely unfulfilled. The document analysis, in fact, did not provide any evidence in this respect, and participants’ comments denote that advocacy and lobbying are regarded only as *potential future* functions for medical regulators.

In view of this and considering that regulators’ intentions and intended roles are often not clear, it is difficult to foresee future developments and provide the regulators with recommendations in this regard.

As a final consideration, overall results indicate that some of the barriers and challenges mentioned by the research participants go beyond the area of direct influence of medical regulatory bodies. For instance, the majority of issues regarding eHealth data collection, analysis, and reporting can be addressed only by data holders such health-care service organizations and government agencies. Also, the lack of data analysis and interpretation capabilities among the medical workforce can be tackled mainly by educational institutions and CPD providers through targeted education and training. Finally, all the enablers listed by the key informants (e.g., use of champions and larger use of AI) can be implemented exclusively at workplace level. Given this, it appears that more research is needed to define and clarify roles and responsibilities of all stakeholders involved. Also, it is evident that all stakeholders will need to be willing to collaborate and coordinate if the intent is that of fostering a cultural shift in CPD requirements, addressing contextual factors, and promoting digital health innovation in the CPD landscape. In view of this, medical regulatory bodies have the opportunity to take a leadership role in addressing the current lack of direction and guidance in this evolving space.

### Strengths and limitations

The strengths of this study include its multimethod design, the comprehensiveness of the document analysis, and the richness and insightfulness of the interviews’ data.

Some of the limitations inherent to document analysis such as insufficient detail and biased selectivity [40] were mitigated by data triangulation with additional grey literature, relevant scholarly literature, and the results of the interviews.

Along with the strengths of this study and the mitigation strategies employed, there are some limitations. This study was restricted to 5 selected countries and related relevant medical bodies, therefore some of the results might not be valid for other jurisdictions. Despite the fact that key informants were purposively selected because of their role in policy development, their views may not necessarily be representative of the official position of medical bodies they work for.

## Conclusion

This study fills a gap in the literature by showing that current medical regulations and CPD requirements do not foster the use of eHealth data for CPD completion and compliance. Further research on key policy concepts, regulators’ internal organisational factors, and interorganisational relationships and dynamics within the CPD ecosystem is needed to formulate jurisdiction-specific recommendations for future policy development.

Results from this study also revealed that medical regulatory and certifying bodies are willing to assume advocacy and lobbying functions to address existing data availability and accessibility issues, but the majority does not foresee changes in policy documentation in order to include the explicit mention of eHealth data usage for CPD compliance.

This study finally shows that the alignment of all relevant stakeholders – including regulatory and certifying bodies, CPD providers, professional associations, healthcare service organisations, governmental agencies – is required to tackle existing barriers and challenges and promote digital health innovation in CPD practices. Moreover, it suggests that medical regulators could fill the current vacuum of leadership in the CPD ecosystem playing a more central role in the fostering of eHealth data usage for CPD completion and compliance.

## Electronic supplementary material

Below is the link to the electronic supplementary material.

Supplementary Material 1

## Data Availability

All documentary data analysed in this study is publicly available on the internet. Deidentified interview transcripts are available from the corresponding author on reasonable request.

## Abbreviations

ABMS: American Board of Medical Specialties
ABO: American Board of Ophthalmology
ABS: American Board of Surgery
ACCME: Accreditation Council for Continuing Medical Education
Ahpra: Australian Health Practitioner Regulation Agency
CFPC: College of Family Physicians of Canada
CME: Continuing Medical Education
CMQ: Collège des médecins du Québec
CPD: Continuing Professional Development
CPSM: College of Physicians and Surgeons of Manitoba
FMRAC: Federation of Medical Regulatory Authorities of Canada
GMC: General Medical Council
MBA: Medical Board of Australia
MCNZ: Medical Council of New Zealand
MFI: Model for Improvement
MRA: Medical Regulatory Authorities
Pii: Practice Improvement Initiative
QI: Quality Improvement
Royal College: Royal College of Physicians and Surgeons of Canada
