# Supplementary material for: The role of medical regulations and medical regulators in fostering the use of eHealth data for strengthened continuing professional development (CPD): a document analysis with key informants’ interviews
Source: BMC Med Educ. 2025 Jul 1;25:871. doi: 10.1186/s12909-025-07443-w (PMC12210532; doi:10.1186/s12909-025-07443-w)
Supplement: Supplementary file 1 — Supplementary Material 1 [file 12909_2025_7443_MOESM1_ESM.docx]

## Additional file 1

## Themes, sub-themes, and representative quotes from the interviews with the key informants

| **Theme** | **Sub-theme** | **Quotes** |
| --- | --- | --- |
| Use of eHealth data for strengthened CPD | Valuable but complex | “I think there's huge potential. […] we'd like to see a world where practitioners are supported to continue to improve and to learn and develop and grow, and data is such a fantastic way for that to happen.” P15, Australia  “I do think we have an opportunity to accelerate this really valuable high quality CPD around practice improvement and more workplace-based, more experiential, more engaging patients, and using these data sources and enabling them.” P06, Canada  “…you know, we just started to wade into these waters and it's a huge and complex issue, and we don't have any real answers at this point in time, more questions than anything, but it's definitely, you know, I think any board would tell you there's value.” P09, USA |
|  | Barriers | “…we actually understood that, that it might be difficult [for medical practitioners] accessing data for some of the things that they need to do, so it's not, we know it's not at the moment readily available. We know this is a work in progress” P13, Australia  “In our jurisdiction, so we can go from they have wonderful data to they have no data, or they don't have data that they make available to either the regulator or the practitioner” P01, Canada  “There is data available about your practice as a doctor in the [jurisdiction], but the integrity and the consistency and the availability of that data varies enormously from location to location and from specialty to specialty and has very significant shortcomings when it comes to national data for non-consultant grade doctors.” P19, UK  “There are many different electronic medical records out there and so there's no standardization with respect to the type of electronic medical records that are being used.” P07, Canada  “Think there's lots of use for the records that we're not getting because they're so locked down. Um, it would be nice to see some sort of centralized system of communicating across platforms.” P10, USA  “…enable like tech standards to be able to reach these records and aggregate and make sense of it in a way that's meaningful for CPD purposes. I think it can be done, but it looks to me like we're a very long way away from that yet.” P06, Canada  “Importantly, a key element of the efficacy of that approach is to provide comparative performance data for them from their peers. And that's hard to do but is really important and really valuable to drive the type of reflection and improvement that you need.” P18, USA  “We have to settle with this legal issue of accessing data from public domain, because they call us public even if we're the order of physicians, and the access of the personal data of patients. We're going to get there. It takes time, but we're going to get there.” P08, Canada  “I think privacy legislation has got a lot of great things about it, but it's also created some challenges, too. I think one of the things that I continually see is people misunderstanding exactly what it is that they can and can't share. And so debunking myths about what's possible, even within the framework of privacy legislation and other legal frameworks is really important.” P15, Australia |
|  | Challenges | “I think it’s just so early in the stage that... and to my knowledge, there are no plans. There’s not even a group that’s looking at it. But there’s been a lot of talk and recommendations, but no one’s actually rolling up their sleeves to move this along. […] There’s no national coordination of this, right? It’s left to like, I think the hospitals… […] I think if we were to get together and say, ‘Here’s the greater good, and here's what it looks like.’ And we paint the picture of what we really want to enable, then we could divide off the roles. […] we would do it in a coordinated way that may actually contribute to a cohesive system. But that’s what’s missing.” P06, Canada  “I think it's, it's potentially problematic because the more you make top-down expectations of individual practitioners, the more cynical they're going to get about how relevant those top-down mandates are for their performance and their improvement.” P18, USA  “Our doctors are extremely busy and there, they’re to some degree cynical about their own professional development. They're burned out and exhausted and they're tired and they just don't want anything more that isn't going to find use to them.” P18, USA  “I think doctors in Australia and overseas have been very cautious about the idea of having some publicly available data that measures their individual practice against the practice of their colleague. I think there's justifiable fear that an unfortunate event, or that's outside of their control, for example, might be publicly disclosed.” P04, Australia  “The whole privacy bit, um, doctors don't tend to trust bureaucracies, and the idea of their personal data, even if you promise hand on heart, that it's going to be de identified, there's some trust that'll need to be built. So, while theoretically fantastic, I suspect it would be one of those things that would be a long-term project and a work in progress and a need to build up a lot of trust in the system.” P13, Australia  “I think the privacy concerns, the concerns about some malpractice issues coming up are real in there. But I do think, on the whole, people are interested in improving their practice and, you know, if that proved to be a valuable mechanism for doing so, you could probably get a lot of large buy-in for that. Um, if they are shared [malpractice] that there's not punitive measures around it.” P09, USA  “You've got to do it in a proper way. And doctors don't know how to, at least older doctors don't know how to do this because this [quality improvement] is a science. They didn't learn this in medical school. Younger doctors know this. So, we have taken it upon ourselves to try to teach them, you know, here's how you do quality improvement.” P11, USA  “…there are some individuals who haven't had any training about improvement or how to use data. And so, some of what needs to happen is educational opportunities for them to learn about, well, what do you do with the data? How do you write an aim? What does it mean to write an aim? How do you narrow what you're doing?” P05, USA |
|  | Enablers | “you've got to take into account the need to form some sort of advisory groups that look at information coming up from physicians […] a variety of educational specialists and mentors, um, who can support teams and individuals look at and work with their data. […] colleagues and peers and facilitators, and also experts who can look at data and help you understand what you might be seeing as an individual practitioner.” P18, USA  “…one of the better solutions that I've heard is really about promoting clinical champions to lead and show the way. Really using early adopters, people who are well respected, well trusted, on the ground, that people work with every day, and getting them engaged so that others see what they're doing and how they're doing it and learn from them. The fears drop away, and the trust is built.” P15, Australia  “…more artificial intelligence and machine learning and truly leveraging that in a way that is enabling and not contributing to burnout and extra work.” P06, Canada |
|  |  |  |
| eHealth data and policy | Current policy choices and future policy directions | “It's actually not for us to say you have to use this set of data.” P13, Australia  “We're not going to mandate it because it's a level of detail too far for us, because our guidance is deliberately set at a very high level. […] So, we won't prescribe it. So, we'd say what data would you find useful and how does that shine a light on your practice, and how does that help you reflect on what you're doing, and you choose the data source that is most appropriate for that activity.” P19, UK  “I would find it unlikely that we would say it must be this way with the electronic health record or with anything because that's sort of what we tried earlier, which really sort of said you must do a QI project. And that ended up being very limiting for the diplomates who were engaged in a lot of different efforts to improve care. I think it's unlikely that we would focus that strongly on electronic health records, because we're moving more towards a wide door approach. We're really trying to encourage and engage physicians in this work in the many levels that they already do and that really encourage more of that.” P05, USA  “We are changing. There is a regulation on medical chart in [country], and the regulation for inside hospital and outside of hospital. We're changing our regulation of medical records outside of hospital, and it's submitted to the [governmental organisation] because we want the regulation to clearly stipulate that every physician in [country] will have to use an EMR. No more of those chart handling, and paper, and everything, and the loss, and the whatever, finished. So yes, we're changing our regulation, and we're working on that right now to be sure that our regulation of chart handling for outside of hospital, for clinical research, for medical practice, or whatever specific specialty, everybody will have to use an EMR. Everybody will have to use an EMR to evaluate their practice. So, they will have to use the tools developed by the companies to evaluate their practice, and put that in the declaration saying, "Yes, I did make an evaluation of my practice using the tool such." So that's why we're doing that.” P08, Canada |
|  | Current policy intent | “And to be perfectly honest […] in the discussions that we had at the CPD expert group that developed the framework [name of framework], people weren't attuned to the idea that there would be this sophisticated analytics. […] That there was recognition that there were these data collected but not a clear sense of how that might be used other than with those certain manual extraction type of way. […] It wasn't in the clear intentions, but the potential was by no means excluded. […] So I think the [regulator, as much as any other group would benefit from being shown what can be done with this.” P03, Australia  “our [name of CPD requirements] isn't premised on having to access that [eHealth data], that we see that as an added bonus, that would be really helpful. Um, but you know, you could do it and we would expect in the first instance, most people would do it on their own data. […] this whole framework is not, is not absolutely dependent. It's assisted, by access to external data, but you can do it without it.” P13, Australia  “we actually want to encourage what we're calling a wide door approach to improving health and health care. […] there are a lot of different ways to systematically work to improve the systems in which you work. And getting data from the electronic health record is one way to do that and it can be a very good way to do that. But it's not the only way to engage in this work.” P05, USA |
|  | Meaning of term “data” | “We think much more broadly around this whole reflecting on outcomes, um, because we are using data in a much broader sense. We're talking about data from multi-source feedback, outcomes data from visits to your practice and, and different things outside the normal thought pattern around data.” P01, Canada  “because of the so many different ways and data sources and data sets that a doctor might use to inform their CPD, we haven't been particularly… well, we haven't been prescriptive at all […] because whether you're a doctor wanting to get your individual data, whether you're seeking unit level service data to improve the service that you work in. [00:23:21] I mean, they're all valid sources of information for doctors.” P16, New Zealand |
|  |  |  |
| Roles and responsibilities of medical bodies |  | “It's our job to form those alliances, form those partnerships with a whole range of different stakeholders, and all be pulling in the one direction to help practitioners to continuously improve.” P4, Australia  “The [participating body] would have to do that [making data accessible to practitioners] through collaboration with jurisdictions primarily, but also, the more it becomes relevant to people working in the broadest application of medicine in [country], it would also involve the [government] and the private health systems and so on.” P03, Australia  “I think there probably is a role for [participating body] encouraging the more consistent application and attribution of electronic health data to individual clinicians. And we could do that through our lobbying function, so we talk to the politicians who run the country and run the departments of health.” P19, UK  “…we want to be able to provide resources for them on how to access data, and how to use that data to create practice improvement goals. […] we have an interest in making sure that our physicians have access to educational resources that support requirements that are mandated by their licensing authority.” P07, Canada  “…one of our recommendations essentially is about rewarding organizations who put a lot of emphasis on using personal performance data to improve the practice based on data in their systems.” P18, USA |
